# Transcriptomics analysis of hulless barley during grain development with a focus on starch biosynthesis

**DOI:** 10.1007/s10142-016-0537-5

**Published:** 2016-12-02

**Authors:** Yawei Tang, Xingquan Zeng, Yulin Wang, Lijun Bai, Qijun Xu, Zexiu Wei, Hongjun Yuan, Tashi Nyima

**Affiliations:** 1 Tibet Academy of Agricultural and Animal Husbandry Sciences, Lhasa, 850002 China; 2 State Key Laboratory of Barley and Yak Germplasm Resources and Genetic Improvement, Lhasa, 850002 China; 3 Agricultural Research Institute, Tibet Academy of Agricultural and Animal Husbandry Sciences, Lhasa, 850002 China; 4 Chengdu Life Baseline Tecshnology Co., LTD, Chengdu, 610041 China; 5 Institute of Agricultural Resources and Environment Science, Tibet Academy of Agricultural and Animal Husbandry Sciences, Lhasa, 850002 China

**Keywords:** Hulless barley, Comparative transcriptome approach, Differentially expressed genes (DEGs), Starch synthesis-related genes (SSRGs)

## Abstract

Hulless barley, with its unique nutritional value and potential health benefits, has increasingly attracted attentions in recent years. However, the transcription dynamics during hulless barley grain development is not well understood. In the present study, we investigated the transcriptome changes during barley grain development using Illumina paired-end RNA-sequencing. Two datasets of the developing grain transcriptomes from two barley landraces with the differential seed starch synthesis traits were generated, and comparative transcriptome approach in both genotypes was performed. The results showed that 38 differentially expressed genes (DEGs) were found co-modulated in both genotypes during the barley grain development. Of those, the proteins encoded by most of those DGEs were found, such as alpha-amylase-related proteins, lipid-transfer protein, homeodomain leucine zipper (HD-Zip), NUCLEAR FACTOR-Y, subunit B (NF-YBs), as well as MYB transcription factors. More interestingly, two genes *Hvulgare_GLEAN_10012370* and *Hvulgare_GLEAN_10021199* encoding SuSy, *AGPase* (*Hvulgare_GLEAN_10033640* and *Hvulgare_GLEAN_10056301*), as well as *SBE2b* (*Hvulgare_GLEAN_10018352*) were found to significantly contribute to the regulatory mechanism during grain development in both genotypes. Moreover, six co-expression modules associated with specific biological processes or pathways (M1 to M6) were identified by consensus co-expression network. Significantly enriched pathways of those module genes showed difference in both genotypes. These results will expand our understanding of the complex molecular mechanism of starch synthesis during barley grain development.

**Electronic supplementary material:**

The online version of this article (doi:10.1007/s10142-016-0537-5) contains supplementary material, which is available to authorized users.

## Introduction

Seed starch, as a primary source of carbohydrate for the human and animal diet, is the major storage compound accumulated in the cereal endosperm and also has been applied in numerous industrial. In recent years, many researches have focused on how to better understand the process of starch synthesis in cereal grains (Morell and Myers 2005, Tomlinson and Denyer 2003, Chetouhi et al. 2016). Currently, different classes of enzyme activities have been identified as being necessary for starch granule synthesis via involved in different pathways, such as ADP-glucose pyrophosphorylase (AGPase), granule-bound starch synthase (GBSS) (Patron et al. 2002), as well as the rather complex amylopectin molecule including soluble starch synthase (SS), starch-branching enzyme (SBE), and starch-debranching enzyme (DBE) (Mutisya et al. 2003). In addition, Benedito et al. have firstly demonstrated that a total of 15,786 genes were differentially expressed during seed maturation in *Medicago truncatula* (Benedito et al. 2008) and found more than 45% of the functionally classified seed-regulated genes were assigned to metabolic pathways, comprised of carbohydrate, amino acid, lipid, energy, and secondary metabolism, indicating that the seed development process is prone to controlled by metabolic pathways. Among metabolites, carbohydrates are well known to represent a major carbon and energy source during seed development. Moreover, sucrose plays a dual role in the cell, as it is central in carbohydrate metabolism and acts as a signal molecule triggering storage-associated processes (Gibson 2005). Recent data has also highlighted a number of transcription factors that are specifically involved in the process of seed development, including B3, MYB, bHLH, and AP2 (Sreenivasulu and Wobus 2013). Taken together, these results illustrate the complexity of seed development regulation involved in seed development. Moreover, several studies have also provided evidence of the concerted action of a complex regulatory network triggering the seed development process (Gutierrez et al. 2007, Weber et al. 2005). In recent years, these mechanisms have been widely studied in the model plants *M. truncatula* (Benedito et al. 2008, Gallardo et al. 2007) and soybean (Dhaubhadel et al. 2007, Jones and Vodkin 2013, Severin et al. 2010). However, there has been limited research regarding gene expression patterns related to starch biosynthesis during barley grain development.

In this study, RNA-seq technology was used to profile transcriptional dynamics during barley grain development of two Tibetan hulless barley landraces Zangqing 2000 (Q) and 08-1127 (C2), with the differential grain starch synthesis traits, and then comparative transcription approach in both genotypes was performed. Co-modulated differentially expressed genes (DEGs) and genotype-specific DEGs were identified and functionally annotated, and their expression levels accumulation in different KEGG pathways were also conducted. We further analyzed the starch synthesis-related genes (SSRGs) in both phenotypes and validated by Quantitative real-time PCR (qRT-PCR). This study provides abundant resources for identification of starch synthesis-related genes required for quality improvement in barley.

## Materials and methods

### Plant materials and RNA isolation

Two elite hulless barley cultivars Zangqing 2000 (Q) and 08-1127 (C2) were conserved by the Tibet Academy of Agriculture and Animal Husbandry Sciences and used for gene analysis associated with seed starch synthesis. Zhangqing2000 (Q) has a higher amylose content (68.5%) and β-glucan content (6.88%) as compared to 08-1127 (C2), which has almost 100% amylose content and 11.23% β-glucan (data collected from 2012 to 2013 in Chengdu). The hulless barley plants were cultivated in test plots and grown under normal conditions in three experimental fields in Chengdu, Sichuan Province of China.

After hulless barley booting, grains of Zangqing 2000 (Q) and 08-1127 (C2) plants at 6, 8, 10, 12, 14, 16, 18, and 20 days after pollination (DAP) for RNA extraction were harvested as described in previous studies (Chen et al. 2014). Each sample consisted of grains from at least five individuals and pooled for each biological replicate. Total RNA samples were prepared using Trizol Reagent (Invitrogene, Nottingham, UK), in three replicates, according to the manufacturer’s instructions. The concentration and quality of RNA samples were determined using a NanoDrop 2000 micro-volume spectrophotometer (Thermo Scientific, Waltham, MA, USA). Equal amounts of RNA from each sample of the identical accessions were pooled to construct two complementary DNA (cDNA) libraries.

### RNA-seq library construction and transcriptome sequencing

Based on manufacturer’s instructions from NEBNext Ultra RNA Library Prep Kits for Illumina (NEB, USA), transcriptome sequence libraries were constructed as follows: messenger RNA (mRNA) was isolated from approximately 5 μg of total RNA, and then fragmented, converted to cDNA and PCR amplified. After PCR amplification, each sample library was quantified and qualified using Agilent 2100 Bioanaylzer and ABI StepOnePlus Real-Time PCR System, respectively. In total, eight paired-end libraries were constructed and 200-bp paired-end reads were generated using Illumina HiSeq™ 2000.

### Reads processing and identification of differentially expressed genes

After filtering the raw data following the data-processing steps, including removal of adapter sequences, reads with more than 10% N, and low-quality sequences (the percentage of low-quality bases of quality value ≤5 is greater than 50% in a read). Clean data were generated and assessed using the Q20, Q30, and GC contents. After preprocessing the RNA-seq data, sequence reads for each tissue were mapped using Bowtie version 0.12.7 (Langmead et al. 2009) and TopHat version 1.2.0 (Trapnell et al. 2009). After alignment, normalized gene-level expression values expressed as fragments per kilobase pair of exon model per million fragments mapped (FPKM) were determined using RSEM version 0.9.3 (Trapnell et al. 2010). Spearman correlation coefficient (SCC) analysis was used to quantify the reproducibility of data between the biological replicates of Zangqing 2000 and 08-1127. SCC was calculated from log10-transformed FPKM values of the expressed genes. The Cor.test functions in R were used for SCC analysis. After calculating gene expression levels, DEGs were then screened by noiseqbio (Tarazona et al. 2012). A corrected *P* value <0.05 was used to screen differentially expressed genes between each set of compared samples. The expression patterns and cluster analysis were conducted by Mev v4.7.4 software with K-Means clustering method and Pearson correlation as distance calculation method, respectively (Saeed et al. 2006). In addition, co-expression modules were identified among those two materials, consensus network analysis of Zangqing 2000 and 08-1127 expression data was implemented by R package WGCNA (Langfelder and Horvath 2008), and modules of highly correlated genes based on their expression profiles were identified. These genes were selected based on coefficient of variation with a threshold of 1 across the different samples, and then transformed FPKM values by log2.

### Gene annotation, functional enrichment and pathway enrichment analysis

To investigate the function of those putative differentially expressed genes in both phenotypes, GO functional categories were assigned to differentially expressed genes based on Gene Ontology Database (http://www.geneontology.org/) and KOBAS software was used to pathway enrichment analysis by testing the statistical enrichment of DEGs in KEGG pathways (Xie et al. 2011), and then their relative graphs were constructed using R script.

### Quantitative real-time PCR validation

To confirm the candidate DEGs identified from RNA-seq assay, 10 SSRGs with great alteration expression levels were chosen and validated by qRT-PCR. The primers employed in the qRT-PCR experiments are designed by Primer 5 and listed in Table S1; hulless barley gene (HvADP) was used as a standard control (Ferdous et al. 2015). qRT-PCR was implemented using the SYBR premix Ex Taq kit (TaKaRa, China) on an ABI 7500 Real-Time System (Applied Biosystems); the procedure was conducted as follows—95 °C for 30 s, 95 °C for 5 s, and 60 °C for 30 s, 40 cycles—and then generated the melt curves for verification of amplification specificity by a thermal denaturing step. The relative quantitative method (2^−△△CT^) was used to calculate the fold change in the expression levels of target genes (Schefe et al. 2006). All reactions were performed in three technical replicates using one biological sample.

## Results

### Overview of transcriptome analysis for hulless barley grains in Zangqing 2000 (Q) and 08-1127 (C2)

**Fig. 1.**
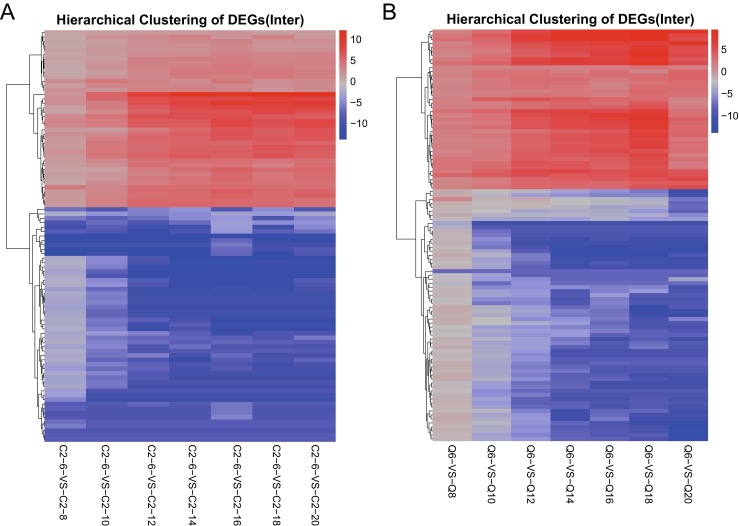
RNA-seq analysis of 08-1127 (C2) and Zangqing 320 (Q) grain dynamic development transcriptome. **a** Hierarchical clustering of normalized expression levels for all global expressed genes shown distinct gene expression profiles in 08-1127 (C2). **b** Hierarchical clustering of normalized expression levels for all global expressed genes showed distinct gene expression profiles in Zangqing 320 (Q)

To identify global expression genes associated with seed starch synthesis in two elite hulless barley cultivars Zangqing 2000 (Q) and 08-1127 (C2), genomics-wide analysis of expression genes was conducted using RNA-seq technology. On average, after the low-quality reads were removed, RNA-seq experiments yielded between 40.11 and 42.02 millions paired-end reads per sample corresponding to over 4.456 to 4.670 billion nucleotides per sample (Table S2). Of those, over 97.5% of the clean reads with high-quality scores at the Q20 level (a base quality greater than 20 and an error probability of 0.01) were identified. GC contents of the clean data were almost identical for Zangqing 2000 (Q) and 08-1127 (C2) grains mRNA libraries (51.73 and 52.43%, respectively). A high proportion of clean reads from C2 (83.59~86.79%) and Q (82.79~88.13%) were readily mapped to barley reference genome sequence, corresponding to C2 (27.68~60.77%) and Q (32.70~62.86%) transcriptome gene set of Tibetan hulless barley. At last, 32,149 global expression genes (almost covered 81.98% of the whole gene set) were identified from total of RNA-seq experiments. After that, the abundance of global expression genes were quantified using Cufflinks (Trapnell et al. 2012) and measured as fragments per kilobase of exon model per million mapped reads (FPKM) (Table S3). In addition, gene expression data showed a higher spearman correlation coefficient (SCC) among biological replicates, indicating high correlation between sequencing replicates in this study (Fig. S1). Moreover, hierarchical clustering of normalized expression levels for all global expressed genes showed distinct gene expression profiles in 08-1127 (C2) and Zangqing 2000 (Q) genotypes (Fig. 1a, b).

### Differentially expressed genes associated with seed starch synthesis traits in both phenotypes

**Fig. 2.**
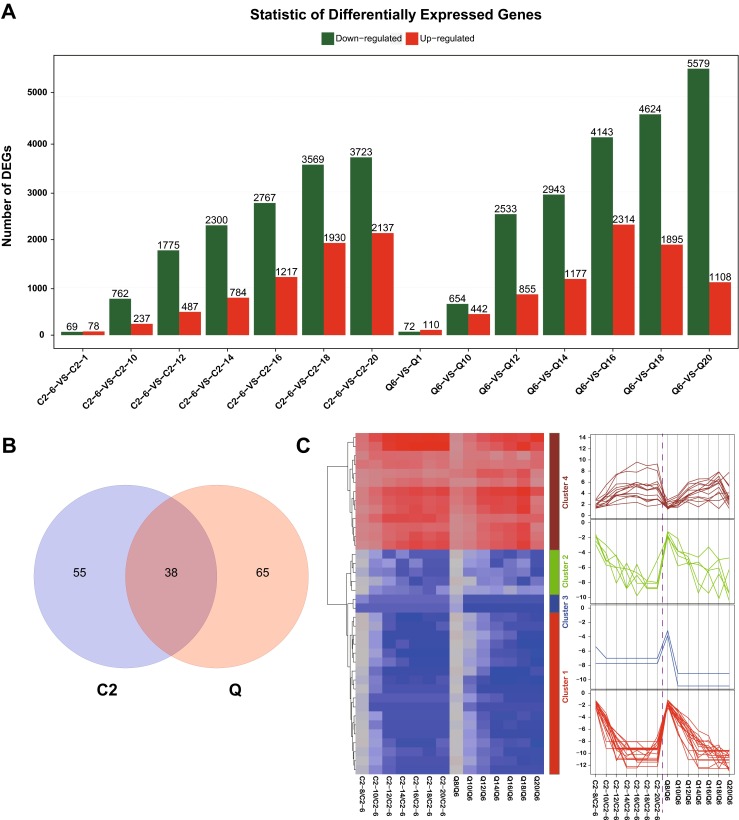
Transcriptional changes of 08-1127 (C2) and Zangqing 320 (Q) for grain dynamic development. **a** Statistic of differentially expression genes including upregulated and downregulated in each comparison groups in 08-1127 (C2) and Zangqing 320 (Q). By performing pair-wise comparisons of Zangqing 320 (Q) and 08-1127 (C2), samples at 6, 8, 10, 12, 14, 16, 18, and 20 days after pollination (DAP), expression changes of all genes between the 6-day group and other days after pollination were analyzed. **b** Venn diagram analysis of common grain development related genes in 08-1127 (C2) and Zangqing 320 (Q) across seven time points (20 vs 6, 18 vs 6, 16 vs 6, 14 vs 6, 12 vs 6, 10vs 6, and 8 vs 6 days). **c** Clustering and heatmap of common differentially expressed genes based on the expression profiles in 08-1127 (C2) and Zangqing 320 (Q)

To investigate DEGs related to seed starch synthesis traits in both phenotypes, transcriptional changes were determined by comparing Zangqing 2000 (Q) and 08-1127 (C2) genotypes grains transcriptomes and DEGs were identified using Noiseq software, respectively (Tarazona et al. 2012). For each hulless barley phenotype, performing pair-wise comparisons of changes in gene expression in distinct samples at 8, 10, 12, 14, 16, 18 and 20 days after hulless barley booting were conducted, compared with 6-day group, respectively. The results showed that majority of seed-regulated genes were downregulated in both phenotypes and a higher percentage of seed starch synthesis-repressed genes was detected in Zangqing 2000 (Q) (Fig. 2a, Table S4). In addition, many genotype-specific DEGs with great alteration were also found; a total of 103 DEGs and 93 DEGs were identified related to seed starch synthesis traits in Zangqing 2000 (Q) and 08-1127 (C2), respectively. Of those, 38 DEGs were co-modulated in both genotypes during barley grain development (Fig. 2b, Table S4). In addition, expression levels of co-modulated DEGs indicating a certain level of conservation DEGs associated with barley grain development. After that, expression changes pattern of co-modulated DEGs in Zangqing 2000 (Q) and 08-1127 (C2) grains are displayed with different colors representing relative gene expression levels using heatmap software. Based on overall gene expression patterns obtained from heatmap, these co-modulated DEGs were divided into four classes assigned to class I, class II, class III, and class IV, respectively. Of those, those genes assigned to class I, class II, and class III showed downregulated expression patterns in both genotypes, while genes belong to class IV were accumulated in both genotypes, comprised of peroxidase, zinc-finger homeodomain protein 1 and nuclear transcription factor Y subunit B-1 (Fig. 2c). Interestingly, some functional genes encoding alpha-amylase related proteins were downregulated expression in class II, along with a diverse sets of transport proteins and transcription factors, such as non-specific lipid-transfer protein (LTP) and Myb transcription factor (Table S5). Further, we focused on co-modulated DEGs and genotype-specific DEGs, which are more likely to play crucial roles in seed development associated with starch biosynthesis in hulless barley.

### Starch synthesis-related genes identified in hulless barley Zangqing 2000 (Q) and 08-1127 (C2)

**Fig. 3.**
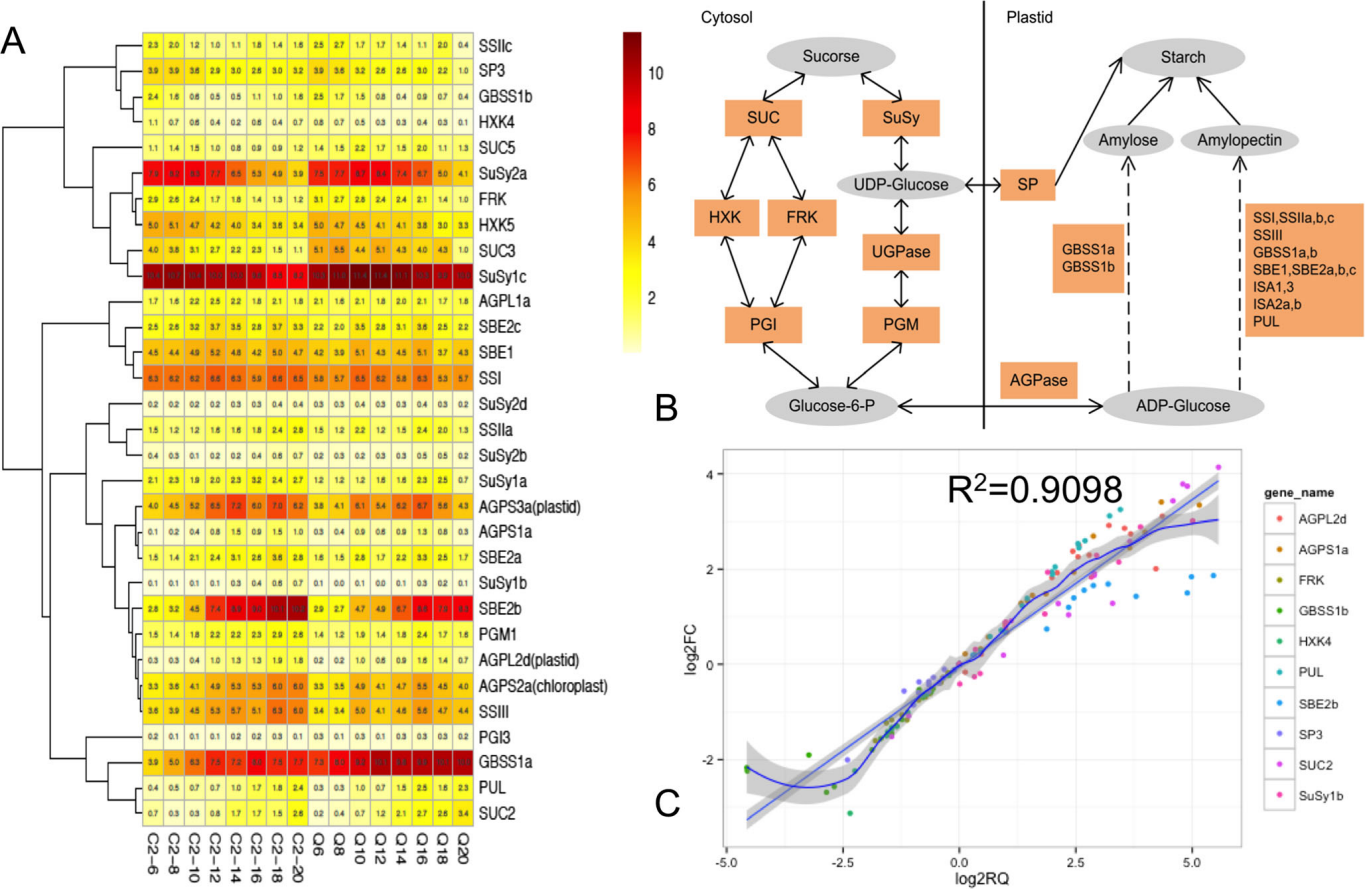
Starch synthesis-related genes (SSRGs) identified in hulless barley Zangqing 320 (Q) and 08-1127 (C2). **a** Heatmap of starch synthesis-related genes (SSRGs) identified in hulless barley. **b** Schemes illustrating the involvement of differing suites of isoforms of starch biosynthetic enzymes. Genes in *yellow* are those that are exclusively identified for SSRGs. *AGPase* ADP-glucose pyrophosphorylase, *F16BP* fructose-1,6-biphosphatase, *HXK* hexokinase, *PFK* phosphofructokinase, *PGI* phosphoglucose isomerase, *PGM* phosphoglucomutase, *SBE* starch-branching enzyme, *SP* starch phosphorylase, *SS* starch synthase, *SuSy* sucrose synthase. The *gray background* denotes substrate (sucrose) and product (starch). **c** Correlation between RNA-seq and qPCR data for SSRGs identified in hulless barley Zangqing 320 (Q) and 08-1127 (C2). Each RNA-seq expression data was plotted against that from quantitative real-time PCR and fit into a linear regression. Both *x-* and *y-axes* were shown in log2 scale and each *color* represented a different gene

To demonstrate these candidate genes related to seed starch synthesis in hulless barley, we performed an ortholog search in barley genomes using the starch biosynthesis genes collected from many other species as bait. Based on the best-hit query sequence using BLASTP, the candidate target proteins with *E* values ≤1e−5 were selected and then classified into the corresponding gene families. In total, more than 237 SSRGs were found in hulless barley Zangqing 2000 (Q) and 08-1127 (C2) (Table S6). Of those, 31 SSRGs encode 12 key regulate enzyme family genes were detected as differentially expressed genes in 08-1127 (C2), compared with Zangqing 2000 (Q), including *ADP-glucose pyrophosphorylase* (*AGPase*), *granule-bound starch synthase* (*GBSS*), *soluble starch synthase* (*SS*), *starch-branching enzyme* (*SBE*), *isoamylase* (*ISA*), *starch phosphorylase* (*SP*), *sucrose synthase* (*SuSy*), and *pullanase* (or *beta-limit dextrinase*; *PUL*) (Fig. 3a, b, Table S7). Moreover, a model of starch synthesis showing enzyme activities in hulless barley was constructed; most of the starch synthesis genes were found gradually activated and upregulated from 6 days to 20 DPA, and sucrose synthesis-related genes followed a similar expression pattern, expect for GBSS1b, which was downregulated from 6 to 14 days, and then upregulated from 14 days to 20 DAP. Of which, those genes encoding *SuSy* (*Hvulgare_GLEAN_10012370* and *Hvulgare_GLEAN_10021199*), *AGPase* (*Hvulgare_GLEAN_10033640* and *Hvulgare_GLEAN_10056301*), as well as *SBE2b* (*Hvulgare_GLEAN_10018352*) were found to significantly upregulate expression during grain development (Fig. 3b). At last, 10 SSRGs with great alteration involved in grain dynamic development were validated using qRT-PCR and the results showed higher consistency with expression profiles of RNA-seq data (Fig. 3c).

### Functional annotation of the most abundant transcripts for genotype-specific DEGs and co-modulated DEGs related to seed starch synthesis traits

**Fig. 4.**
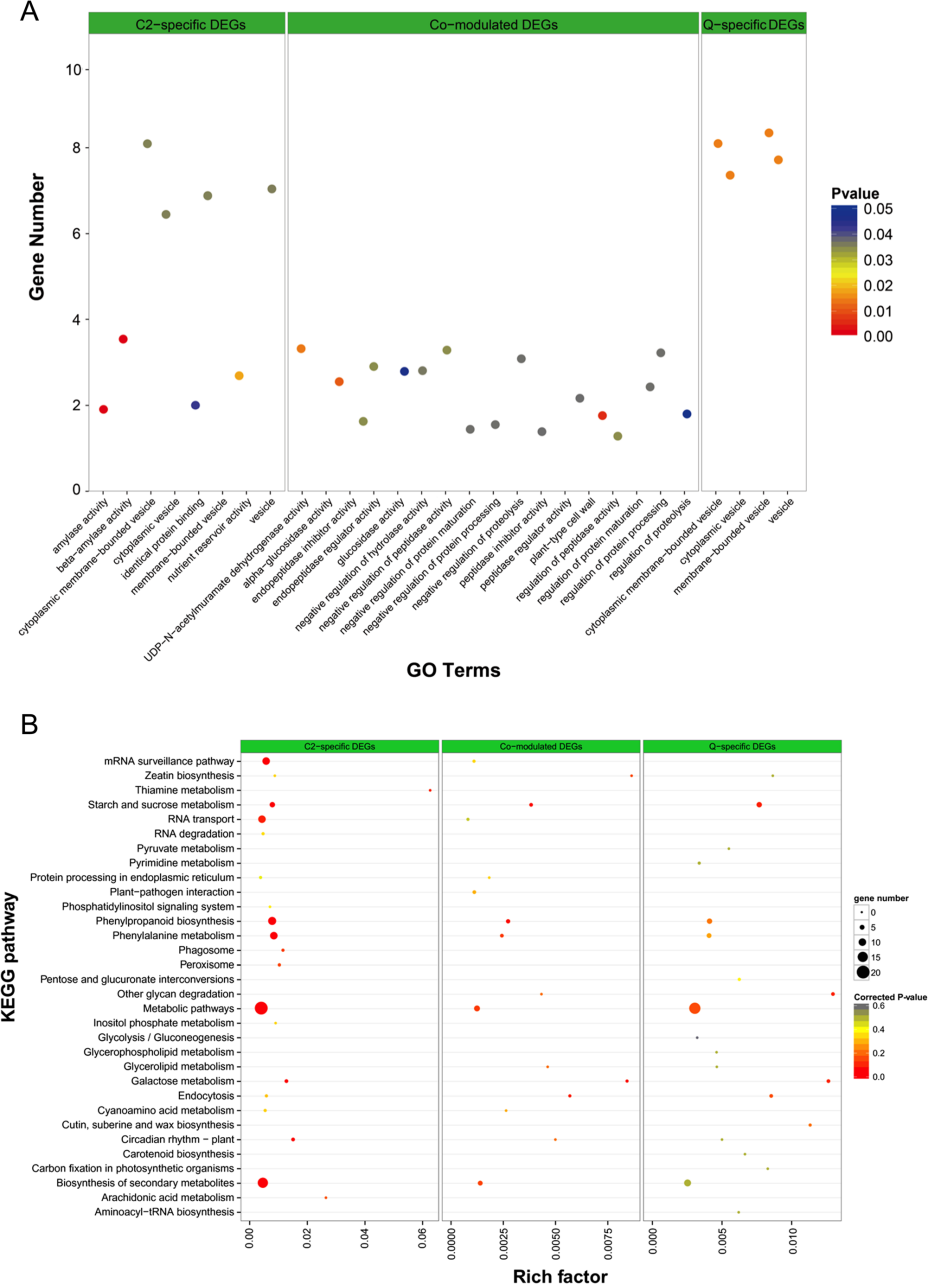
Cross-comparison of functional enrichment analysis among differentially expressed genes (DEGs) including genotype-specific DEGs and co-modulated DEGs in developing grains. **a** Cross-comparison of enriched GO terms among differentially expressed genes in hulless barley dynamic grain development. **b** Cross-comparison of pathway enrichment analysis among differentially expressed genes in hulless barley dynamic grain development

To understand the regulatory mechanisms related to seed starch biosynthesis, consensus co-expressed gene sets were identified based on their transcript profiles of both genotypes using weighted gene co-expression network analysis (WGCNA) (Langfelder and Horvath 2008). Of those, 11,086 genes that fulfilled by filter criteria fell into six co-expression modules (M1 to M6), which were comprised of specific biological processes or pathways that would be involved in seed starch biosynthesis, ranging from 86 (M6) to 4732 (M1) genes. The GO terms which comprised of “plant-type cell wall,” “alpha-glucosidase activity,” “UDP-N-acetylmuramate dehydrogenase activity,” “endopeptidase inhibitor activity,” and “endopeptidase regulator activity” were commonly over-represented in both genotypes. In addition, a relatively larger portion of specific DEGs in hulless barley 08-1127 (C2) were found to be significantly enriched in almost 18 GO terms, especially most of DGEs related to those molecular functions, such as “beta-amylase activity,” “amylase activity,” “nutrient reservoir activity,” “cysteine-type endopeptidase activity,” “cysteine-type peptidase activity,” as well as “electron transport chain.” However, only few portion of specific DEGs in hulless barley Zangqing 2000 (Q) was found significantly enriched, such as “extracellular region” (Fig. 4a, Table S8). In addition, to further analyze the biological functions of co-modulated and genotype-specific DEGs, pathway enrichment analysis of those DEGs was implemented by KOBAS. Of those, five significantly enrichment pathways for hulless barley 08-1127 (C2) genotype-specific DEGs were identified, including mRNA surveillance pathway, phenylpropanoid biosynthesis, phenylpropanoid metabolism, metabolic pathway, as well as RNA transport. However, only one significantly enrichment pathways (metabolic pathway) was identified for hulless barley Zangqing 2000 (Q) genotype-specific DEGs. Moreover, no significantly enrichment pathway was detected for co-modulated DEGs for both phenotypes (Fig. 4b, Table S9).

## Discussion

Hulless barley, with its unique nutritional value and potential health benefits, has gained significant attention in recent years. To develop the better hulless barley cultivars with desirable dietary characteristics, it is significantly necessary to the exploitation of hulless barley germplasm resources and to reveal the molecular mechanism of grain development in hulless barley, especially seed starch biosynthesis (Akpinar et al. 2015). In this study, RNA-seq technology was used to profile the grain dynamics development of two Tibetan hulless barley landraces Zangqing 2000 (Q) and 08-1127 (C2), with the differential grain starch synthesis traits. Totally, 32,149 global expression genes (almost covered 81.98% of the whole gene set) were identified from RNA-seq profiles. For each hulless barley phenotype, a comparison of changes in gene expression between 6 DAP and other DAP were conducted. Here, 103 DEGs and 93 DEGs with great alteration were identified associated with dynamics hulless barley grain development in Zangqing 2000 (Q) and 08-1127 (C2), respectively. Of those, 38 DEGs were co-modulated in both genotypes during grain development. More interestingly, some functional genes encoding alpha-amylase-related proteins, along with a diverse set of transport proteins and transcription factors, such as non-specific lipid-transfer protein (LTP) and Myb transcription factor, were identified downregulated in both phenotypes, respectively, while peroxidase (POD), zinc-finger homeodomain protein 1, and nuclear transcription factor Y subunit B-1 were found upregulated in both phenotypes. Alpha-amylase (EC 3.2.1.1) has been recently described as the “best known amylolytic enzyme” in plants (Da Lage et al. 2013). Currently, four α-amylase categories from HvAMY1 to HvAMY4 have been identified and demonstrated to be expressed at different grain developmental stages in barley (Radchuk et al. 2009). In this study, *AMY2a* (*Hvulgare_GLEAN_10006031*), *AMY2b* (*Hvulgare_GLEAN_10007167*), *AMY2d* (*Hvulgare_GLEAN_10001214*), and *AMY3a* (*Hvulgare_GLEAN_10028893*) were found accumulated in both phenotypes and upregulated from 6 days to 20 DPA. In addition, NUCLEAR FACTOR-Y, subunit B (NF-YBs), also known as Heme Activator Protein 3 (HAP3) or CCAAT-Binding Factor A (CBF-A), has been reported as important regulators of numerous in plant developmental in plants (Liang et al. 2012, Janská et al. 2014). It has been proposed that LTPs play an essential role in transport of cuticular lipids through plant cell walls (Kader 1996). Moreover, HD-Zip IV TFs were found to be associated with differentiation and maintenance of outer cell layers, and regulation of lipid biosynthesis and transport. Additionally, previous study indicated that HD-Zip might interact with various MYB factors in different cell types to enable diverse functions (Suo et al. 2003). So we speculated that HD-Zip might interact with various MYB factors to the regulation of lipid biosynthesis and transport, together with lipid-transfer proteins (LTPs). Previous study suggested that the majority of sucrose results from storage lipid degradation and does not from other soluble sugars within the Arabidopsis seeds (Huber et al. 2007); it is identical to the results from germinating barley seeds (Jia et al. 2013). Subsequently, sucrose may be resolved into glucose and fructose by SUSY (sucrose synthase), and then enter glycolysis. In our study, two genes *Hvulgare_GLEAN_10012370* and *Hvulgare_GLEAN_10021199* encoding SuSy were found. It is reported that, Susy, as a highly regulated enzyme that reversibly converts sucrose and nucleoside diphosphate into the corresponding nucleoside diphosphate glucose and fructose (Baroja-Fernández et al. 2012). We further studied the transcripts involved in the synthesis of main storage nutrient in hulless barley grain. As we all know, starch biosynthesis in the barley grains requires the coordinated activities of several core enzymes (Radchuk et al. 2009). Among them, *AGP-S1a* (*Hvulgare_GLEAN_10056301*) and *AGP-L2d* (*Hvulgare_GLEAN_10033640*) were mainly expressed in the developing grain at high levels in both hulless barley phenotypes, suggesting their importance at the first step of starch biosynthesis. The upregulation of these related genes contributes to the gradual accumulation of starch. Next, the chain elongation of amylose and amylopectin are distinctively catalyzed by the starch granule-bound form of starch synthase (GBSS) and soluble form of starch synthase (SS), respectively. For starch synthase (GBSS), *GBSS1a* (*Hvulgare_GLEAN_10032543*) showed much higher expression level in 08-1127 (C2) than Zangqing 2000 (Q), contrary to *GBSS1b* (*Hvulgare_GLEAN_10049996*). *GBSSIa* may act as the main limiting enzyme in the endosperm amylose production. These results are consistent with previous research in wheat and rice (Hirose and Terao 2004, Vrinten and Nakamura 2000). However, the expression levels of *SSs* in 08-1127 (C2) and Zangqing 2000 (Q) were not significantly differential expression in our study. Furthermore, the *SSs* gene may not play typical roles in the elongation of amylopectin chains during starch biosynthesis in barley. In addition, *SBE2b* (*Hvulgare_GLEAN_10018352*) and *PUL* (*Hvulgare_GLEAN_10034352*) were also found significant upregulated in both phenotypes, but the expression levels were lower in 08-1127 (C2) than Zangqing 2000 (Q). Comprehensively, *AGP-S1a*, *AGP-L2d*, *GBSSIa*, *SBE2b*, and *PUL* may significantly affect the starch biosynthesis through mainly expressed in barley grain (Fig. 3c). However, in our previous study, most of those genes including *AGPase*, *GBSS*, *SS*, SBE, *ISA,* and *PUL* were found to show no significant difference between two other barley accessions (Chen et al. 2014). In starch biosynthetic pathway, each enzyme plays a distinct role, but presumably functions as part of a complex network. In this synthesis network, genes controlling amylopectin and amylose synthesis possibly interact (Fulton et al. 2002, van de Wal et al. 1998). Therefore, we speculated that starch synthesis trait of these two accessions with the different percentage of amylose might be mediated by multiple genes that involved in complex pathway (Fig. 3b). Singletary et al. reported that numerous pleiotropic effects on *SS*, *SBE*, and *AGPP* resulting from mutations in genes were observed for specific enzymes of the pathway (Singletary et al. 1997). Tetlow et al. (2004) also demonstrated that the formation of multi-enzyme complexes is also supported by direct evidence for interactions between starch biosynthetic enzymes in wheat endosperm. Subsequently, high molecular weight fractions isolated from developing maize and wheat endosperm revealed the existence of enzyme complexes comprising of SSI, SSIIa, SSIII, SBEIIa, and/or SBEIIb in various combinations (Hennen-Bierwagen et al. 2008, Tetlow et al. 2008). Partial purification has also revealed that the SSIII-containing complex also contained SSIIa, SBEIIa, and SBEIIb, and large and small subunits of AGPP and pyruvate phosphate dikinase (PPDK) were also identified using proteomic analysis (Hennen-Bierwagen et al. 2009). These findings suggested that a broader metabolic significance of these enzyme complexes might involve in complex network to affect the starch biosynthesis.

## Electronic supplementary material

Figure S1. SCC analysis of the mRNA data for 08–1127 (C2) and Zangqing 320 (Q) using log10-based FPKM values. The hierarchical clustering dendrogram was inferred according to SCC analysis results. (EPS 1943 kb).

ESM 1 High resolution image (PNG 160 kb).

ESM 2 (XLSX 63559 kb).
