# Supplementary figures and images for: Transcriptomics analysis of hulless barley during grain development with a focus on starch biosynthesis
Source: Funct Integr Genomics. 2016 Dec 2;17(1):107–17. doi: 10.1007/s10142-016-0537-5 (PMC5203864; doi:10.1007/s10142-016-0537-5)

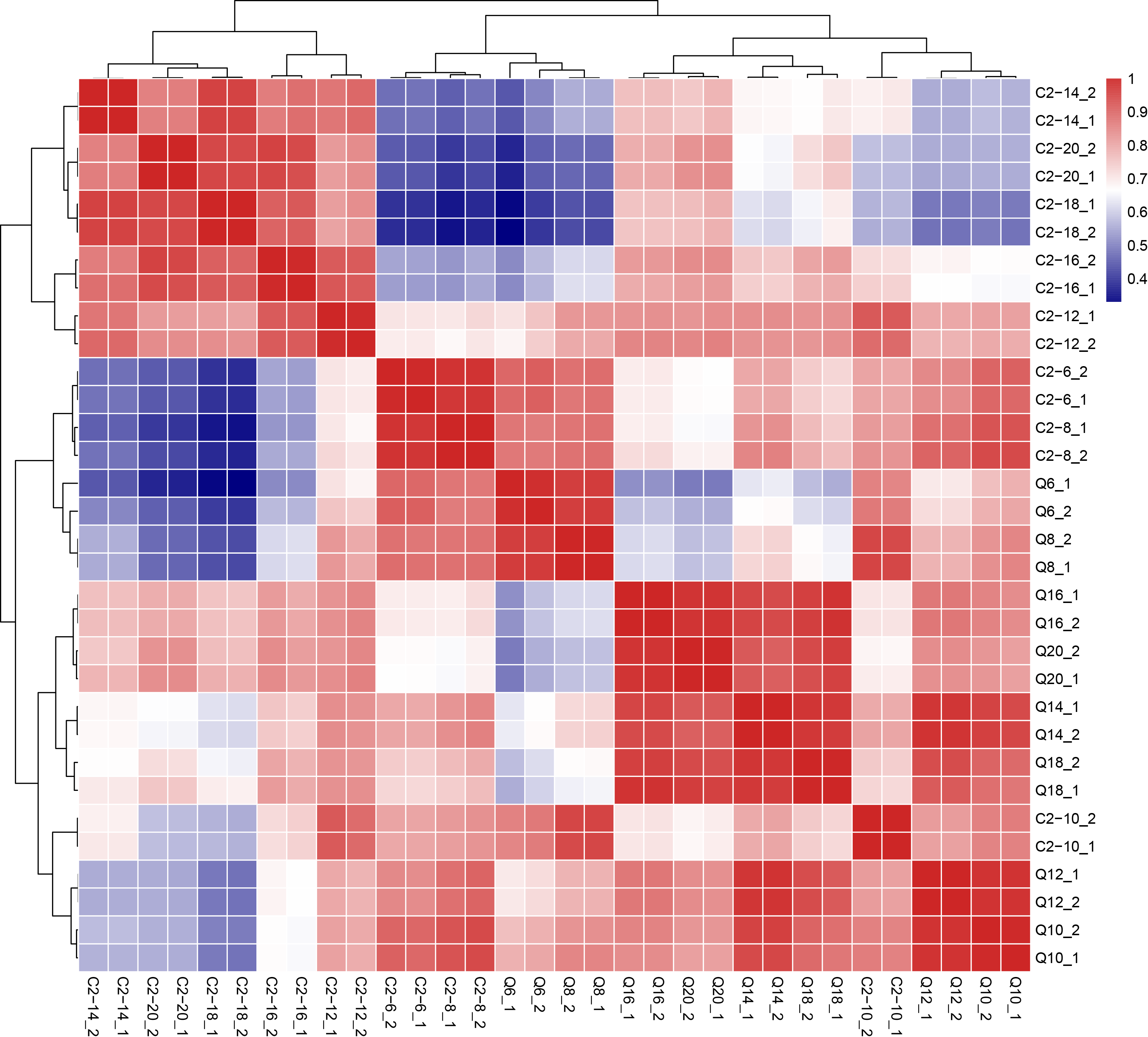

Supplement: Supplementary file 2 — High resolution image (PNG 160 kb). [file 10142_2016_537_Fig5_ESM.png]
